# Targeted Transcriptional Analysis of IgA Vasculitis, IgA Nephropathy, and IgA-Dominant Infection-Related Glomerulonephritis Reveals Both Distinct and Overlapping Immune Signatures

**DOI:** 10.34067/KID.0000000000000123

**Published:** 2023-04-08

**Authors:** Vanderlene L. Kung, Rupali Avasare, Marcia A. Friedman, Stephanie Mengden Koon, Tanaya L. Neff, Sara Protzek, Christopher Corless, Victoria Krajbich, Naly Setthavongsack, Rebecca Ditmore, Randall Woltjer, Nicole K. Andeen

**Affiliations:** 1 Department of Pathology and Laboratory Medicine, Oregon Health & Science University, Oregon Health & Science University, Portland, Oregon; 2 Department of Medicine, Division of Nephrology and Hypertension, Oregon Health & Science University, Portland, Oregon; 3 Department of Medicine, Division of Rheumatology, Oregon Health & Science University, Portland, Oregon; 4 Department of Dermatology, Oregon Health & Science University, Portland, Oregon; 5 Knight Diagnostic Laboratories, Knight Cancer Institute, Oregon Health & Science University, Portland, Oregon

**Keywords:** glomerular and tubulointerstitial diseases, calprotectin, IgA dominant infection related glomerulonephritis, IgA nephropathy, IgA vasculitis, IL9, KIR, MRSA, nanostring, Staphylococcus aureus, transcriptome

## Abstract

**Key Points:**

- Skin IL-9, calprotectin, and KIR gene expression may be predictive of subsequent kidney involvement in patients with IgAV.
- Histologically similar patients with IgAN, IgAV, and IgA-IRGN can be distinguished by their immune transcriptomes.
- Kidney biopsies from patients with IgA-IRGN are enriched for transcripts involved in granulocyte chemotaxis.

**Background:**

IgA vasculitis (IgAV), IgA nephropathy (IgAN), and IgA-dominant infection-related glomerulonephritis (IgA-IRGN) have shared histopathologic features, but differences in clinical management and prognosis. The most serious IgAV organ involvement is in the kidneys (IgAV nephritis). In this study, we hypothesized that targeted immune transcript profiling could aid in (*1*) predicting the development of IgAV nephritis in patients with cutaneous IgAV and (*2*) differentiating IgAN, IgAV, and IgA-IRGN.

**Methods:**

RNA was extracted from 24 formalin-fixed paraffin-embedded tissue specimens (16 kidney, 8 skin) from 21 patients with IgAV nephritis (n=7), IgAN (n=5), and IgA-IRGN (n=4), and IgAV skin biopsies from patients with (n=3) and without (n=5) IgAV nephritis. Differential gene expression and gene set enrichment analysis were performed on a total of 594 transcripts (Nanostring immunology panel) profiled using the nCounter system.

**Results:**

Skin biopsies in patients with IgAV who develop kidney involvement exhibit reduced *S100A8/S100A9*, *IL9*, and killer cell immunoglobulin-like receptor expression. The kidney tissue immune transcriptomes of IgAN, IgAV, and IgA-IRGN are largely overlapping. IgA-IRGN kidney biopsies are, however, uniquely enriched for transcripts involved in granulocyte chemotaxis.

**Conclusion:**

This study identifies immune transcript signatures that may predict IgAV nephritis in skin biopsies and distinguish IgA-IRGN from IgAN and IgAV in kidney biopsies.

## Graphical abstract

**Figure:**
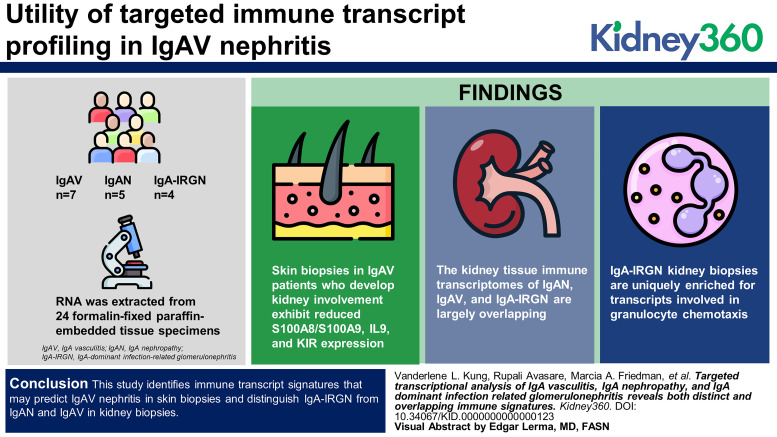

## Introduction

IgA is the most abundant immunoglobulin isotype and is paramount to the maintenance of mucosal immunity. The pathologic deposition of IgA in tissue is shared by three related but distinct clinical diagnoses: IgA vasculitis (IgAV), IgA nephropathy (IgAN), and IgA-dominant infection-related glomerulonephritis (IgA-IRGN). There can be considerable overlap in the clinical presentation of these three disease entities, and their morphologic differentiation may be diagnostically challenging; however, the accurate classification of diseases with aberrant IgA tissue deposition is necessary because there are significant differences in treatment and prognosis among these diseases.

IgAN is the most common glomerulonephritis worldwide.^1^ It is frequently preceded by or concurrent with upper respiratory tract infections and is characterized by dominant or codominant IgA deposition in the glomerular mesangium and a variety of proliferative features by light microscopy.^2,3^ In contrast to IgAN, which is renal limited, IgAV, also known as Henoch-Schönlein purpura, is a systemic small vessel vasculitis that is the most common small vessel vasculitis in children and adolescents.^4^ Because most patients with IgAV (95%) present with a symmetric, dependent-area predominant purpuric rash, the histologic diagnosis of IgAV is most frequently made on skin biopsy, on the basis of the finding of leukocytoclastic vasculitis (small vessel wall necrosis and neutrophilic inflammation) and IgA-dominant immune deposits in small vessels on immunofluorescence microscopy. IgAV nephritis, which is morphologically indistinguishable from IgAN, is the most serious long-term manifestation of IgAV, occurring in approximately 40% of children with IgAV and accounting for approximately 1%–2% of all childhood end stage kidney disease,^5^ and while adults are less commonly affected, adults with IgAV nephritis are significantly more likely to have severe renal outcomes.^6^ Why only some patients with IgAV develop renal involvement is incompletely understood, and thus, the first aim of this study was to use targeted transcriptional profiling in skin biopsies from patients with IgAV to identify immune signatures associated with the subsequent development of kidney involvement.^7,8^

A third pathology characterized by IgA deposition in tissue is IgA-IRGN. IgA-IRGN commonly affects older male patients with underlying comorbidities, such as diabetes mellitus, alcoholism, and hypertension, and is associated with ongoing, apparent, or occult bacterial (frequently *Staphylococcus aureus*) infection. Thus, in contrast to IgAN and IgAV, where active disease is treated with immunosuppression, the first line in the management of IgA-IRGN is the identification and treatment of underlying infection. The kidneys are the primary site of pathology in IgA-IRGN, but approximately 20% of patients develop an infection-related rash, which clinically and histopathologically mimics IgAV.^9,10^ IgA-IRGN is diagnosed on renal biopsy and can often be distinguished from IgAN and IgAV by the presence of exudative features, significant glomerular C3 deposition, lack of IgA staining in sclerotic glomeruli,^11^ and hump-shaped deposits in mesangial notch regions on electron microscopy^12–14^; nevertheless, certain cases remain difficult to classify, particularly in patients without known infection or in the setting of cutaneous rash. Thus, the second aim of this study was to determine immune transcripts that distinguish IgA-IRGN from IgAV and IgAN.

## Methods

After obtaining IRB approval, 24 archived formalin-fixed paraffin-embedded tissue blocks (16 kidney, 8 skin) from 21 patients were used, including kidney biopsies from patients with IgAV with renal involvement (Henoch-Schönlein purpura/IgAV nephritis) (n=7), IgAN (n=5), and IgA-IRGN (n=4) (Table 1). In addition, skin biopsies were obtained from patients with IgAV with skin-limited disease (n=5) and from patients with IgAV with both skin and subsequent kidney diseases (n=3). Neutrophils, lymphocytes, and eosinophils were counted in six 400× fields, three perivascular and three interstitial, for each routine H&E-stained IgAV skin biopsy by a pathologist blinded to clinical data, and paired means between patients with and without subsequent kidney involvement were compared using the Wilcoxon signed-rank test.

**Table 1:** Clinical features at the time of biopsy

Case	Tissue Available	Diagnosis	Age	Sex	Serum Creatinine (mg/dL)	Proteinuria	Hematuria	Steroid Therapy Prior to Biopsy	Additional Pertinent History
IgAV1	Kidney	IgAV	Adult	M	Normal	Subnephrotic	Yes	No	Leukocytoclastic rash and complements and serologies normal
IgAV2	Kidney	IgAV	Adult	M	AKI	Nephrotic range	Yes	Yes	Leukocytoclastic rash, diabetes, and HTN
IgAV3	Kidney	IgAV	Pediatric	M	0.2	Nephrotic syndrome, uPCR 26.5 g/g	Yes	Yes	Leukocytoclastic rash and complements and serologies normal
IgAV4	Kidney	IgAV	Pediatric	F	0.5	uPCR 2.7 g/g	Yes	No	HTN and complements and serologies normal
IgAV5	Kidney and skin	IgAV	Pediatric	M	0.4	uPCR 1.4 g/g	Yes	Yes, before kidney but not skin biopsy	Abdominal pain, leukocytoclastic rash, and complements and serologies normal
IgAV6	Kidney and skin	IgAV	Adult	F	0.7	uPCR 3.9 g/g	Yes	Yes, before kidney but not skin biopsy	Leukocytoclastic rash and complements and serologies normal
IgAV7	Kidney and skin	IgAV	Pediatric	F	0.7	uPCR 1.4 g/g	Yes	Yes, before kidney but not skin biopsy	Leukocytoclastic rash and complements and serologies normal
IgAV8	Skin	IgAV	Pediatric	F	Normal	No	No	No	Leukocytoclastic rash
IgAV9	Skin	IgAV	Pediatric	F	Normal	No	No	No	Leukocytoclastic rash
IgAV10	Skin	IgAV	Adult	M	Normal	No	No	No	Leukocytoclastic rash
IgAV11	Skin	IgAV	Pediatric	M	Normal	No	No	No	Leukocytoclastic rash
IgAV12	Skin	IgAV	Pediatric	M	Normal	No	No	No	Leukocytoclastic rash
IgAN1	Kidney	IgAN	Pediatric	F	1.8	2+	Yes	No	Concurrent viral pneumonia
IgAN2	Kidney	IgAN	Pediatric	M	1.1	uPCR 2.7 g/g	Yes	No	Complements and serologies normal
IgAN3	Kidney	IgAN	Adult	F	1.1	Yes	Yes	No	Complements and serologies normal
IgAN4	Kidney	IgAN	Adult	M	Normal	Yes	Yes	No	NA
IgAN5	Kidney	IgAN	Adult	M	4.0	6.5 g/d	Yes	No	HTN and complements and serologies normal
IgA-IRGN1	Kidney	IgA-IRGN	Adult	F	6.1	Yes	Yes	No	Rheumatoid arthritis; MRSA septicemia with abscesses; positive ANA (1:320), RF, atypical p-ANCA, and cryoglobulin (1 of 2); and negative hepatitis B and C
IgA-IRGN2	Kidney	IgA-IRGN	Adult	F	1.9	Nephrotic syndrome, 8.0 g/d	Yes	No	NASH cirrhosis; bacterial peritonitis; MRSA bacteremia; positive ANA (1:80) and RF; low C3 and C4; and other serologies and cryoglobulins negative
IgA-IRGN3	Kidney	IgA-IRGN	Adult	M	AKI (dialysis)	Yes	Yes	No	Skin/soft-tissue infection with *Staphylococcus aureus*
IgA-IRGN4	Kidney	IgA-IRGN	Adult	M	AKI (dialysis)	Yes	Yes	No	Skin/soft-tissue polymicrobial infection, diabetes, HTN, and serologies and cryoglobulins negative

Adult age >18 years. Pediatric age ≤18 years. IgAV, IgA vasculitis; HTN, hypertension; uPCR, urine protein-to-creatinine ratio; IgAN, IgA nephropathy; NA, not available; IgA-IRGN, IgA-dominant infection-related glomerulonephritis; MRSA, methicillin-resistant *Staphylococcus aureus*; RF, rheumatoid factor; NASH, nonalcoholic steatohepatitis.

Formalin-fixed paraffin-embedded blocks were cut at 10 microns×15 sections; kidney samples were collected as curls while on sections from skin, the superficial dermis and epidermis were enriched by macrodissection away from the deep dermis. Collected tissue was deparaffinized, and total nucleic acids were extracted using the Macherey-Nagel NucleoSpin Tissue Kit. The samples were DNA-depleted using the ArcticZymes Heat&Run gDNA Removal Kit, followed by RNA quantitation using a Qubit Fluorometer. An RNA 6000 Nano assay was run on an Agilent 2100 bioanalyzer to confirm RNA quality, and for all total RNA samples, >50% of fragments were >200 nucleotides in length (DV200 mean±SD, 75%±9%).

The nCounter human immunology panel was used to profile 594 targets. nSolver defaults were used to filter on the basis of default quality control measures. Data are deposited in GEO (GSE220100). Transcripts were imported into the R/Bioconductor package DESeq for all downstream analyses. Transcripts that summed to less than one count across all samples were removed. Principal component analysis (PCA), followed by permutation ANOVA (999 permutations) implemented using the adonis algorithm from the R/Bioconductor *vegan* package, was used to test for significant separation between patients. Differential expression analysis was performed with sex included as a covariate in the DESeq2 model. Significance was defined as false discovery rate–adjusted *P* < 0.05 and fold change >2.00. Gene set enrichment analyses were performed using the R/Bioconductor *gage* package to test hallmark gene sets in the Molecular Signatures Database and gene ontology terms in the gene ontology hierarchies biological process, molecular function, and cellular component. Potential outlier samples were identified by performing robust PCA statistics with *PcaGrid* and calculating sample orthogonal and score distances with *pcaDiagplot*. All differential gene expression and gene enrichment analyses were reperformed with potential outlier samples dropped out to verify that results were not driven by potential outliers.

### Ethics Approval and Consent to Participate

This study was performed with OHSU IRB (FWA00000161; IRB00000471) approval and in accordance with the Declaration of Helsinki.

## Results

### Immune Transcript Signatures Cluster by Tissue Type

**Figure 1:**
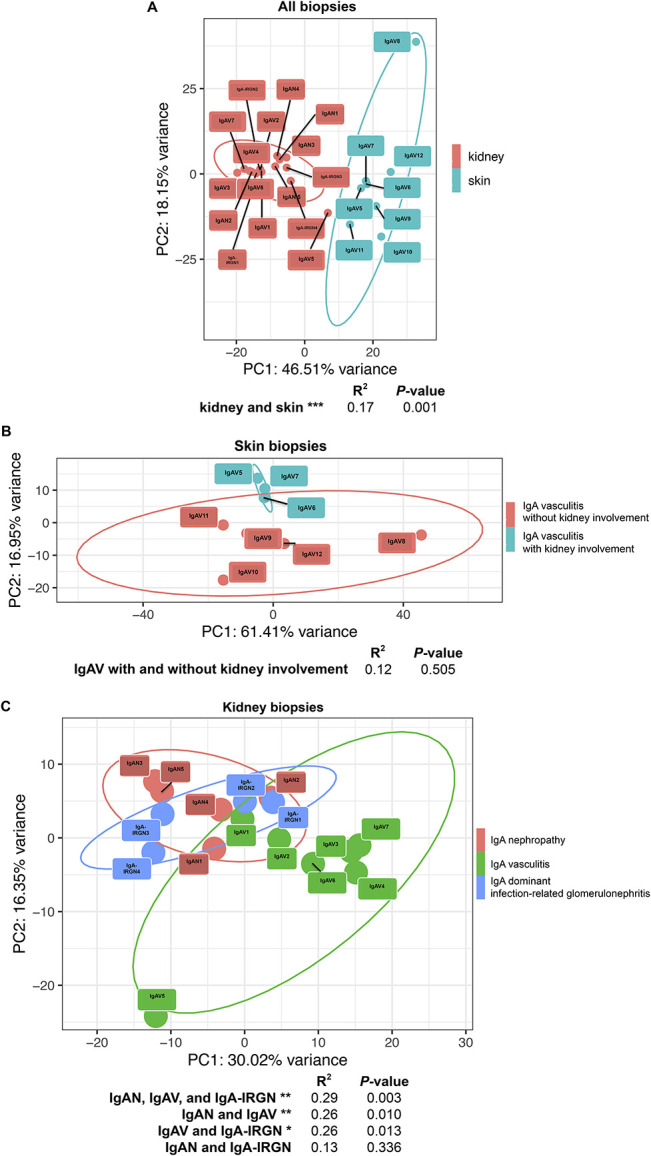
**Principle component analysis of 594 immune targets in IgAV skin biopsies and IgAN, IgAV, and IgA-IRGN kidney biopsies.** (A) Skin and kidney biopsy transcript profiles are distinct in PCA space. (B) Within skin biopsies, transcript profiles from patients with IgAV with (IgAV5–7) and without (IgAV8–12) renal involvement cannot be resolved. (C) Within kidney biopsies, IgAN (IgAN1–5) and IgA-IRGN (IRGN1–4) overlap in PCA space and IgAV (IgAV1–7) is separate from both IgAN and IgA-IRGN in PCA space. The *x* axis represents the PC1, and the *y* axis represents the PC2. Ellipses correspond to the 95% confidence ellipse. Patient designations are as assigned in Table 1. R^2^ and *P*-values are from adonis variance analysis (*P < 0.05, **P < 0.01, ***P < 0.001). IgAV, IgA vasculitis; IgAN, IgA nephropathy; IgA-IRGN, IgA-dominant infection-related glomerulonephritis; PC, principal component; PCA, principal component analysis.

PCA using the normalized counts of all 594 assayed immune transcripts demonstrated robust separation of kidney and skin samples (Figure 1A). As expected, irrespective of whether patients developed IgAV nephritis, IgAV skin biopsies cluster more closely with each other than with any kidney biopsies. Similarly, IgAV nephritis biopsies are closer in PCA space to other kidney biopsies with aberrant IgA deposition than to IgAV skin biopsies.

### Skin biopsies in patients with IgAV who develop renal involvement exhibit reduced *S100A8/S100A9*, *IL9*, and killer cell immunoglobulin-like receptor (KIR) expression.

**Figure 2:**
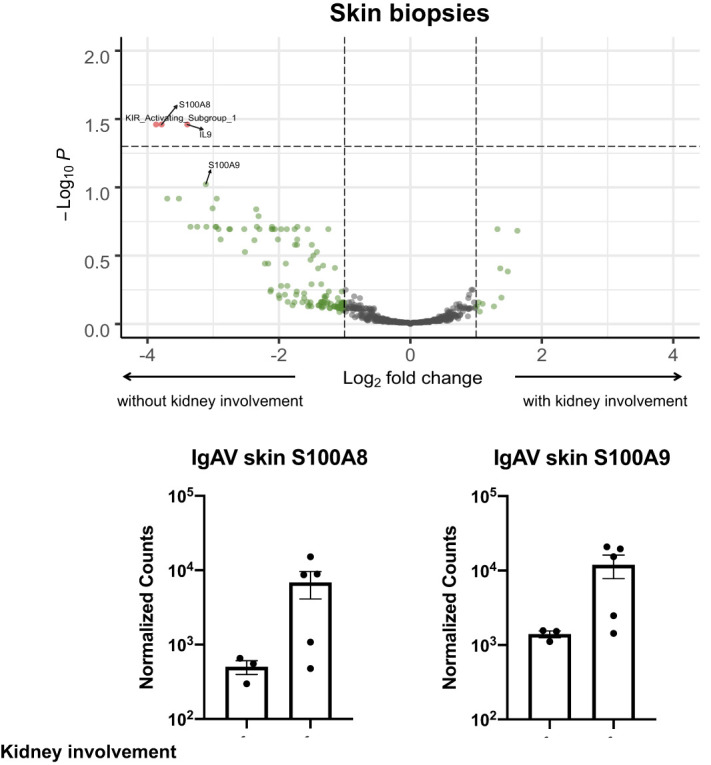
***S100A8/S100A9* is among a limited number of genes significantly differentially expressed between IgAV skin biopsies with and without subsequent renal involvement.** Means with standard error of the mean are displayed. See also Supplemental Table 1. IgAV, IgA vasculitis.

IgAV skin biopsies from patients with and without subsequent renal involvement were histologically indistinguishable, demonstrating a leukocytoclastic vasculitis with IgA deposition and no significant difference in the number of infiltrating perivascular or interstitial neutrophils, lymphocytes, and eosinophils (data not shown). No patients received any immunomodulatory treatments before skin biopsy. Although IgAV skin biopsies from patients with and without subsequent kidney involvement did not significantly separate in PCA space (Figure 1B), differential gene expression analysis yielded three targets (Figure 2 and Supplemental Table) significantly decreased in expression in skin biopsies from patients who developed IgAV nephritis. The first, *S100A8*, along with *S100A9*, encodes calcium-binding proteins that heterodimerize to form calprotectin, an abundant myeloid cell cytoplasmic protein. The second transcript significantly depressed in skin biopsies from patients who develop IgAV nephritis encodes the cytokine IL-9, which is produced by T lymphocytes and type 2 innate lymphoid cells. As with calprotectin, IL-9 has context-dependent function in regulating autoimmunity^15^; because with calprotectin, IL-9 has context-dependent functions in regulating autoimmunity. Finally, the most highly differentially depressed skin transcripts in patients who develop IgAV nephritis were detected with the KIR Activating Subgroup 1 probe, which hybridizes to the *KIR3DS1*, *KIR2DS1*, *KIR2DS2*, and *KIR2DS4* transcripts that encode activating and inhibitory receptors regulating NK-cell effector function.^16^

### Kidney biopsies from patients with IgA-IRGN are enriched for transcripts involved in granulocyte chemotaxis.

**Figure 3:**
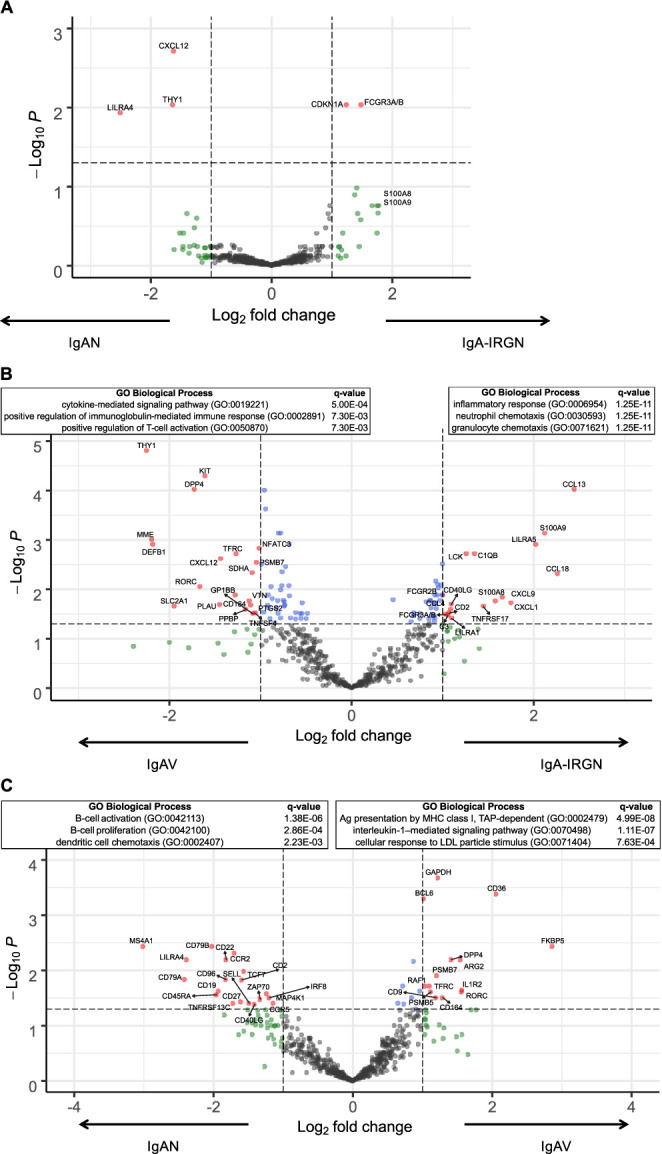
IgAV nephritis, IgAN, and IgA-IRGN have distinct and overlapping immune signatures. Differential gene expression analysis between IgAN and IgA-IRGN (A), IgAV and IgA-IRGN (B), and IgAV and IgAN (C). The top three most significantly enriched GO biological processes for each of the differentially expressed gene sets are listed. See also Supplemental Table. GO, gene ontology; IgAV, IgA vasculitis; IgAN, IgA nephropathy; IgA-IRGN, IgA-dominant infection-related glomerulonephritis.

IgAN, IgAV, and IgA-IRGN kidney biopsies were histopathologically matched as well as possible and demonstrated predominantly mesangial deposition of IgA, generally with C3, with a similar degree of glomerular activity (endocapillary hypercellularity and crescents) and chronic injury in glomeruli and the tubulointerstitium, which were minimal to mild in most patients (Table 2). Compared with either IgAV or IgAN, IgA-IRGN was enriched for *S100A9* and *S100A8* gene targets and *FCGR3A/B*, encoding the low-affinity Fc receptor, Fc*γ*RIII (CD16) (Figure 3, A and B). Additional targets significantly increased in expression in IgA-IRGN in comparison with IgAV include those encoding the antimicrobial chemokine CXCL9 (MIG), the neutrophil recruiting and activating chemokine CXCL1, and complement proteins C1q and C3, which are critical to both bacteria opsonization and potentiation of phagocytosis (Figure 3B).

**Table 2:** Pathologic features of kidneys

Case	Diagnosis	M	E	S	T	C	TIN	ATN	Global Gs (%)	IF/TA (%)	IF	EM Deposit Location
1	IgAV nephritis	1	0	0	0	2	N	Y	0	10	IgA: 3−4+, C3: 3−4+	Mesangial
2	IgAV nephritis	0	1	0	0	1	N	Y	0	10	IgA: 3−4+, C3: 3+	Mesangial and subendothelial
3	IgAV nephritis	1	1	0	0	1	N	Y	0	0	IgA: 4+, C3: 1+	Mesangial and subendothelial
4	IgAN	0	1	0	0	2	N	Y	0	0	IgA:4+, C3: 3+	Mesangial and intramembranous
5	IgAN	1	1	1	0	1	Y	N	0	15	IgA: 4+, C3: 1−2+	Mesangial
6	IgAN	1	1	1	1	1	Y	N	11	30	IgA: 2−3+, C3: 3−4+	Mesangial, subendothelial, and rare subepithelial
7	IgAN	1	1	1	0	1	Y	N	20	25	IgA: 4+, C3: 3+	Mesangial and subendothelial
8	IgAN	0	1	1	1	1	N	Y	25	30	IgA 3+, C3: 2+	Mesangial
9	IgA-IRGN	Y	Y^a^	N	0	Y	N	N	30	30	IgA and C3 codominant	Mesangial, subendothelial, and subepithelial
10	IgA-IRGN	Y	Y^a^	N	1	N	N	N	10	10	IgA and C3 codominant	Mesangial and subendothelial
11	IgA-IRGN	Y	Y^a^	N	0	Y	Y	Y	19	20	IgA and C3 codominant	Mesangial and subendothlial
12	IgA-IRGN	Y	Y^a^	N	0	Y	Y	Y	14	20	IgA and C3 codominant	Mesangial, subendothelial, and rare subepithelial
13	IgAV nephritis	1	1	0	0	1	N	N	0	0	IgA: 3+, C3: 0	Mesangial and subendothelial
14	IgAV nephritis	0	1	0	0	1	N	Y	0	0	NA	Mesangial
15	IgAV nephritis	0	0	0	0	1	N	N	0	0	IgA:3+, C3: Trace	NA
16	IgAV nephritis	0	0	1	0	1	N	N	0	10	IgA: 3−4+, C3: 1+	Mesangial

TIN, tubulointerstitial nephritis; ATN, acute tubular necrosis; IgAV, IgA vasculitis; IgAN, IgA nephropathy; IgA-IRGN, IgA-dominant infection-related glomerulonephritis; NA, no glomeruli in the sample.

a Exudative features/glomerular neutrophilic influx.

With the exception of these increased innate immune transcripts in IgA-IRGN, the IgAN and IgA-IGRN immune signatures were otherwise remarkably similar, with only five transcripts (3 increased and 2 decreased, Figure 3A) significantly differentially expressed between IgAN and IgA-IRGN. No differences in transcript levels of *CD89*—encoding the IgA Fc receptor expressed on myeloid cells and also released in serum—were detected between IgAN, IgAV, and IgA-IRGN (Supplemental Table).

### Kidney biopsies from treated IgAV nephritis show reduction in B and T-cell transcripts and increased expression of steroid-responsive genes.

Although selected IgAN, IgAV nephritis, and IgA-IRGN kidney biopsies all demonstrated a similar degree of glomerular histologic activity (Table 2), five of the seven patients with IgAV nephritis were treated with steroids before kidney biopsy, whereas none of the patients with IgAN or IgA-IRGN received immunosuppressive therapy before biopsy. Transcriptional analysis revealed IgAV nephritis to be distinct from both IgAN and IgA-IRGN in PCA space (Figure 1C), and differential gene expression analysis revealed 34 transcripts (15 increased and 19 decreased, Figure 3C) significantly differentially expressed between IgAV nephritis and IgAN and 36 transcripts (19 increased and 17 decreased, Figure 3B) significantly differentially expressed between IgAV nephritis and IgA-IRGN. Gene set enrichment analyses on these differentially expressed transcripts identified reduced B and T-lymphocyte activation in IgAV nephritis. One of the most highly overexpressed transcripts detected in IgAV nephritis encodes FK506-binding protein 51, a target known to be potently induced by corticosteroids.^17^ These findings suggest that the IgAV nephritis immune signature in this study was influenced by the effects of corticosteroid therapy before kidney biopsy, and immunologic activity in the IgAV nephritis post-treatment state may be incompletely captured by traditional histologic classifications of activity.

## Discussion

In this study, we performed the first comparison of IgAN, IgAV, and IgA-IRGN by targeted transcriptional profiling and identified immune signatures that shed light on the pathogenesis of these three entities. Novel findings include the identification of transcripts in IgAV skin biopsies that may predict the development of subsequent IgAV nephritis, namely *S100A8/9* (calprotectin), *IL9*, and transcripts encoding KIR proteins. Second, we demonstrated that, compared with IgAN or IgAV, the immune transcriptome of IgA-IRGN is enriched for transcripts encoding proteins involved in granulocyte chemotaxis, including S100A8/9 (calprotectin), FCGR3A/B, CXCL9, CXCL1, C1q, and C3.

Within the skin, the finding of increased dermal expression of transcripts encoding the immunomodulatory proteins IL-9 and calprotectin in patients with skin-limited IgAV points to the fine balance between immune activation and dampening in autoimmunity. Neither calprotectin nor IL-9 encoding transcripts have been previously identified in transcriptional studies comparing IgAV with normal skin and different inflammatory skin diseases.^18^ Calprotectin comprises over half of the soluble cytoplasmic protein content of neutrophils; is also expressed by activated keratinocytes, macrophages, and endothelial cells; and is released by secretion or cell death. Both calprotectin and IL-9 exhibit context-dependent immune activating and dampening functions.^19–28^ A larger validation study set is necessary to determine whether tissue or serum IL-9 and calprotectin levels are predictive of subsequent kidney involvement.

Perhaps the most intriguing finding within the study of IgAV skin is that the KIR transcripts detected with the KIR Activating Subgroup 1 probe (*KIR3DS1*, *KIR2DS1*, *KIR2DS2*, and *KIR2DS4*) are the most highly differentially depressed skin transcripts in patients with IgAV who develop nephritis. KIRs are transmembrane glycoproteins expressed on the surface of NK cells and a subset of T cells that transduce either activating or inhibitory effector functions by interacting with MHC class I molecules and other ligands to regulate immune tolerance. Because there were no differences in expression of MHC class I genes (HLA-A, HLA-B, HLA-C) or NK/cytotoxic lymphocyte markers (CD56 [NCAM1], KLRF1 [NKp80], PRF1, GZMA/B/K, KLRD1 [CD94], IL2RB [CD122], TBX2) between IgAV skin biopsies with and without nephritis (Supplemental Table), individuals with IgAV who develop nephritis likely have reduced KIR gene content and/or transcription compared with those with IgAV without nephritis. Akin to studies of HLA allele frequencies, determining the KIR genotypes of individuals with IgAV may be informative as while some KIR genes display marked allelic variance, others are highly conserved in sequence and are not present in all individuals.^16^ KIR genes have not been previously identified in studies of IgAV pathogenesis, but have been shown to affect susceptibility to ANCA vasculitis^29^ and affect disease progression and treatment response in viral infections, malignancies, and autoimmune diseases.^16^

Within the kidneys, IgA-IRGN is enriched for transcripts involved in granulocyte chemotaxis, particularly *S100A8* and *S100A9*, encoding calprotectin, and *FCGR3A/B*, encoding the low-affinity Fc receptor, Fc*γ*RIII (CD16). These transcriptional-level calprotectin findings are corroborated by protein-level findings from a mass spectrometry-based analysis of the proteomes of IgAN and *Staphylococcus* infection-associated glomerulonephritis, showing the two proteomes to be largely shared, with S100A9 as one of few proteins more abundant in *Staphylococcus* infection-associated glomerulonephritis.^30^ Fc*γ*RIIIa is expressed by macrophages and NK cells, where it binds monomeric IgG attached to target cells to facilitate antibody-dependent cell-mediated cytotoxicity and is upregulated in peripheral blood leukocytes during *S. aureus* infection.^31^ The pathogenesis of IgA-IRGN is hypothesized to involve deposition of preformed, circulating bacterial antigen/antibody complexes and/or *in situ* immune complex or complement deposition against bacterial antigens or glomerular antigens with molecular mimicry.^32^ Although these may be driven by systemic immune responses and have systemic manifestations (ie, cutaneous vasculitis), our findings also support the role of specific, local immune responses within the kidney to influence disease.

A primary weakness of this study is small sample size. No appropriate IgAV or IgA-IRGN datasets are present in GEO to confirm our findings, and the building of a validation cohort is necessary. Steroid treatment before kidney biopsy in IgAV was clinically appropriate, but to some extent confounded our comparison of IgAV nephritis with IgAN and IgA-IRGN. Reassuringly this identified transcriptional changes related to steroid exposure previously largely only described in *in vitro* studies. Additional study constraints that temper interpretation of findings are that transcriptional profiling was limited to the 594 targets within the human immunology panel, and there was insufficient material to also perform quantitative RT-PCR on differentially expressed genes. In addition, because there are histologic compartment-specific differences in cell types and function within the skin and kidneys, further resolution may have been achieved by performing tissue microdissection before transcriptional analysis.

In summary, we identify a cutaneous gene expression signature in IgAV that may predict the subsequent development of kidney involvement—specifically *S100A8/9* (calprotectin), *IL9*, and KIR genes—which lays groundwork for future studies to identify skin biomarkers predictive of IgAV kidney involvement. Our transcriptional findings support prior studies showing mechanistic overlap among IgAN, IgAV, and IgA-IRGN and additionally demonstrate a distinct immunologic milieu in IgA-IRGN composed of granulocyte chemotaxis–related gene signaling, including S100A8/9 (calprotectin), FCGR3A/B, CXCL9, CXCL1, C1q, and C3. These implicate kidney-specific (in addition to systemic) immunologic and complement-mediated effectors in development and propagation of IgA-IRGN and contribute toward the development of biomarkers to separate IgA-IRGN from morphologic mimics in diagnostically challenging cases.

## Supplementary Material

SUPPLEMENTARY MATERIAL

## Data Availability

The datasets used during the current study will be deposited in Gene Expression Omnibus (GEO) and are available from the corresponding author on reasonable request.

## Supplemental Material

Supplemental Table. Complete results of differential gene expression analysis.
